# Co-sensitization between legumes is frequently seen, but variable and not always clinically relevant

**DOI:** 10.3389/falgy.2023.1115022

**Published:** 2023-03-16

**Authors:** Mark Smits, Kitty Verhoeckx, André Knulst, Paco Welsing, Aard de Jong, Marco Gaspari, Anna Ehlers, Paulien Verhoeff, Geert Houben, Thuy-My Le

**Affiliations:** ^1^ Department of Dermatology/Allergology, University Medical Center Utrecht, Utrecht University, Utrecht, Netherlands; ^2^ Center for Translational Immunology, University Medical Center Utrecht, Utrecht, Netherlands; ^3^ Risk Analysis for Products in Development, Netherlands Organisation for Applied Scientific Research (TNO), Utrecht, Netherlands; ^4^ Julius Center for Health Sciences and Primary Care, University Medical Center Utrecht, Utrecht, Netherlands; ^5^ Fresh Food & Chains, Wageningen Food & Biobased Research, Wageningen University, Wageningen, Netherlands; ^6^ Research Centre for Advanced Biochemistry and Molecular Biology, Department of Experimental and Clinical Medicine, Magna Graecia University of Catanzaro, Catanzaro, Italy

**Keywords:** legume allergy, co-sensitization, allergens, protein fractions, seed storage proteins, 2S albumins, 7S globulins, 11S globulins

## Abstract

**Background:**

Food allergy to peanut and soybean, both legumes, is highly prevalent. The consumption of other legumes and legume protein isolates, some of which may be considered novel foods, is increasing. This may lead to an increase in sensitization and allergy and may pose a risk for legume-allergic (e.g. peanut and soybean) patients due to cross-reactivity.

**Objective:**

This study investigated the frequency of co-sensitization and co-allergy between legumes and the role of different protein families.

**Methods:**

Six legume-allergic patient groups were included: peanut (*n *= 30), soybean (*n *= 30), lupine (*n *= 30), green pea (*n *= 30), lentil (*n *= 17), bean (*n *= 9). IgE binding to total extracts, protein fractions (7S/11S globulin, 2S albumin, albumin), and 16 individual proteins from 10 legumes (black lentil, blue lupine, chickpea, faba bean, green lentil, pea, peanut, soybean, white bean, and white lupine) was measured by line blot

**Results:**

Co-sensitization varied from 36.7% to 100%. Mono-sensitization was only found in soybean (16.7%), peanut (10%), and green pea-allergic (3.3%) patients. A high frequency of co-sensitization between the 7S/11S globulin fractions of all 10 legumes and individual 7S and 11S globulins was observed. In peanut and soybean-allergic patients, co-allergies for other legumes were uncommon (≤16,7%), while in green pea, lupine, lentil, and bean-allergic patients co-allergy for peanut (64.7%–77.8%) or soybean (50%–64.7%) was frequently seen.

**Conclusion:**

Co-sensitization between legumes was high, but generally not clinically relevant. Co-allergy to other legumes was not often seen in peanut- and soybean allergic patients. The 7S and 11S globulins were likely responsible for the observed co-sensitization.

## Introduction

Sustainable alternative dietary protein sources are needed as the current livestock production and consumption of meat-based products has a negative impact on resources such as water, agricultural land, and the environment (1, 2). Legumes can be an attractive protein source, because they are rich in protein, fibre, vitamins and minerals (3). The use of legumes and (concentrated) legume protein isolates from pea, lentil, soybean, lupine, chickpea, and beans as alternatives to meat-based proteins has increased due to growing environmental issues and health concerns of consumers (4, 5). An increase in the consumption of legume and legume protein (isolate) derived novel foods potentially increases the prevalence of sensitization and allergy to these foods. Additionally, cross-reactivity of legume proteins may elicit allergic complaints in already legume-allergic (e.g., peanut and soybean) populations.

Sensitization and allergy to multiple legumes in legume-allergic patients were investigated in some previous studies (6–9). Jensen et al. reported a high frequency of co-sensitization for lupine, soybean, pea, alfalfa, mung bean, broad bean, and azuki bean in 10 peanut-allergic patients (8). Additionally, co-sensitization and co-allergy of lupine in peanut-allergic patients has been established in other studies (10–12). Previous studies chiefly focused on co-sensitizations and not on co-allergies. Furthermore, studies were mainly performed in peanut-allergic patients. In addition, the number of investigated legumes was often limited and the number of included patients small. Moreover, studies mainly investigated the co-sensitization between (commercial, easily soluble protein) extracts of different legumes and little is known about co-sensitization at the level of protein families and individual proteins. These protein families contain seed storage proteins such as the 7S (vicilin-type) and 11S (legumin-type) globulins which belong to the cupin family and the 2S albumins which belong to the prolamin family (13, 14). Sensitization to seed storage proteins was found to be an important diagnostic marker for allergy (15).

It is established that peanut, soybean and lupine allergy are among the most prevalent food allergies and therefore require mandatory labelling (16). Because the consumption of other legumes is increasing, it is important to investigate the frequency of co-sensitization and co-allergy between these legumes and assess if it may pose a risk for already legume-allergic patients. The primary objective of this study was to investigate the frequency of co-sensitization and co-allergy for 10 different legumes (peanut, soybean, green pea, chickpea, blue and white lupine, black and green lentil, and white and faba bean) in 6 legume-allergic patients groups (peanut, soybean, green pea, lupine, lentil, and bean-allergic patients). A secondary objective was to investigate which protein fractions (7S/11S globulin, 2S albumin, and albumin) and individual proteins are responsible for co-sensitization.

## Methods

### Patient selection

A representative randomized group of 30 adult patients visiting the Allergology outpatient clinic at the University Medical Center Utrecht with a legume allergy for peanut, soybean, green pea, lupine, lentil or bean were included in the study. When this number was not reached, all available allergic patients were included. Selected patients were preferably diagnosed by a positive oral food challenge or by a convincing history combined with a positive immunoglobulin E (IgE) test in blood (>0.35 kU/L, ThermoFisher, Uppsala, Sweden). An overview of the details of the included patients can be found in Supplementary Table S1.

### Preparation and isolation of legume extracts, protein fractions, and individual proteins

Non-processed and heat processed extracts and protein fractions from peanut (Arachis hypogaea), soybean (Glycine max), green pea (Pisum sativum), chickpea (Cicer arietinum), blue lupin (Lupineus angustifolius) and white lupine (Lupineus albus), black lentil (Lens culinaris) and green lentil (Lens culinaris puyensis), and faba bean (Vicia faba) and white bean (Phaseolus vulgaris).were made, as extensively described previously (17). The protein fractions, 7S/11S globulins (salt soluble globulins), 2S albumin (alcohol soluble prolamins), albumin (water soluble albumins) were extracted using the Osbourne extraction in a procedure adapted from Freitas et al. (18). In short, the globulin fraction was collected by solubilizing defatted legume meal with a high salt buffer (100 mM TRIS/HCl, 1 M NaCl, 10 mM EDTA and 10 mM EGTA, pH 8.2) and centrifuging. The supernatant was collected afterwards which contained the globulins that dissolved in the high salt buffer. By using 100 kDa ultra-filtration, the supernatant was divided in a 7S and 11S globulin fraction (the retentate) and the 2S albumin (the permeate) fraction. Individual seed storage proteins from peanut (Ara h 1, Ara h 2, Ara h 3, Ara h 6), soybean (Gly m 5 and Gly m 6), blue lupine (*α*-conglutin, *δ*-conglutin, and Lup an 1), green pea (pea albumin 1, pea albumin 2, Pis s 1 and legumin A), and white bean (phaseolin and legumin) were prepared as described in Smits et al. (17). Recombinant peanut allergen Ara h 7.0201 (Acc. no. B4XID4) was provided by EUROIMMUN.

### Line blot

The isolated extracts, protein fractions, and individual proteins were placed on a EUROLINE strip (EUROIMMUN, Lübeck, Germany) specially designed for this study by EUROIMMUN. Sensitization was assessed according to the standard manufacturer's instructions. The EUROLINE intensity units (EL) were evaluated using the EUROLineScan software and cross-reactive carbohydrate determinants (CCD) positive sera were reanalyzed after inhibition with Anti-CCD Absorbent (EUROIMMUN). An intensity of 3 (class 1) or higher was rated as positive.

### LC-MS analysis of protein fractions

20 µg of the 2S albumin and 7S/11S globulin fractions were diluted to 0.5 mg/ml in 100 mM Tris (pH 8.0) (40 µl in total). 8 µl of SDS 2.5% (final concentration 0.42% w/v) was added to the samples and the proteins were subjected to reduction, alkylation, tryptic digestion and strong cation exchange StageTip purification as previously described (19). Overnight proteolysis was achieved by adding 400 ng of trypsin per 20 µg of sample (E:S ratio of 1:50). The albumin fractions were processed in a similar way but were diluted to 0.5 mg/mL during the first step in HPLC-grade water instead of 100 mM Tris (pH 8.0) and 500 mM Tris was added to the 2.5% SDS solution that was added to the samples before reduction with DTT. NanoLC-MS/MS analysis was performed on an Easy LC 1,000 nanoscale liquid chromatography system (Thermo Fisher Scientific) coupled to a Q-Exactive mass spectrometer (Thermo Scientific) as previously described with small changes (20). Four microliters (100 ng) of the protein fractions were loaded at a 500 nl/min flow rate onto the analytical column, and peptides were eluted through the reversed-phase column *via* a 45-minute linear gradient. The MS data were processed using Proteome Discoverer v.1.4 (Thermo Scientific) and SEQUEST as search engine. Proteins were identified by searching the mass spectrometric data against the Uniprot Fabaceae database (434,155 entries) accessed on September 2017. Protein hits based on 2 successful peptide identifications (filtered by Percolator, FDR < 0.01) were considered valid.

### SDS-PAGE analysis

15 µl (1 µg/ml) of extract or protein fraction was mixed with 5 µl of 4 × Laemmli sample buffer (BioRad, Hercules, CA) supplemented with DTT. The samples were heated for 5 min at 95°C and shortly vortexed afterwards. 5 µl of Precision Plus Protein Dual Color Standards marker (BioRad, Hercules, CA) and 10 µl of the protein sample were loaded into the wells of an Any kD™ Mini-PROTEAN® TGX™ Precast Protein Gel (BioRad, Hercules, CA) in a buffer tank (Bio-Rad Laboratories Mini-PROTEAN Tetra Cell™) filled with 1 × TGS (BioRad, Hercules, CA). The gels were stained overnight on a plate shaker with InstantBlue™ (Coomassie) gel staining (Expedion, United Kingdom) and then washed with demineralized water for at least 1 h and an image of the gel was made (ChemiDoc™ MP).

### Inhibition assay

A subpopulation of peanut, green pea and lentil-allergic subjects was selected for the inhibition assays. To study the capacity of different 2S albumin and 7S/11S fractions to inhibit IgE binding, sera from selected patients were pre-incubated with unprocessed 2S albumin or 7S/11S fractions of peanut, green pea and lentil. Briefly, 1, 10 and 100 µg/ml of the respective fractions were added to 1:10 (EUROLINE washing buffer) diluted sera and incubated at room temperature for 30 min on an orbital shaker (300 rpm). Subsequently, a line blot was performed. The same protein extracts where used for preparing the line blots and performing the inhibition assay. Pre-incubation with cow's milk extract served as negative control. The positive controls comprised of pre-incubation with the same fraction IgE (self-inhibition) and patients who did not show self-inhibition were excluded from analysis. Inhibition was calculated as percentage with respect to the not inhibited measurement.

### Definitions

Co-sensitization occurs when different IgE antibodies are produced by the patient that bind to proteins but are not necessarily targeted at common structural features (21). In contrast, cross-reactive IgE binding is characterized by IgE antibodies that bind structurally homologous proteins that share common epitopes and cross-reactivity can specifically be determined by inhibition assays (21). Likewise, co-allergy is defined by the presence of clinical allergic complaints for two or more sources, and cross-allergy is defined as the presence of clinical allergic complaints elicited by cross-reactive IgE binding to multiple sources.

### Data analysis

Descriptive analyses were performed to report the frequency of (co-)sensitization and (co-)allergy using SPSS Statistics 25 (IBM Corporation, Armonk, NY, United States). Additionally, the odds ratios (OR) were calculated after adjustment by the Haldane correction to assess the association between legume sensitization and allergy. The Basic Local Alignment Search Tool (BLASTP) was used to investigate the percentage identity. Matches of greater than 50% identity indicated potential cross-reactivity (22).

## Results

### High co-sensitization rates between 10 legumes in different legume-allergic groups

The frequency of sensitization for 10 different legumes (peanut, soybean, green pea, chickpea, blue/white lupine, black/green lentil, faba/white bean) in six legume-allergic patient groups (peanut *n *= 30, soybean *n *= 30, green pea *n *= 30, lupine *n *= 30, lentil *n *= 17 and bean *n *= 9) is shown in Table 1. In all 6 legume-allergic patients groups, co-sensitization was seen for each of the tested legume with a percentage of at least 36.7%. In the bean-allergic patient group, the frequency of co-sensitization for other legumes was the highest (between 77.8% and 100%), while in the soybean and peanut-allergic patient groups lower rates were found (36.7%–76.7%). The percentages in Table 1 indicated that when patients were allergic to green pea, lupine, lentil or bean, they were most likely sensitized to other legumes as well (ranging from 58.8%–100%). In contrast, patients allergic to peanut or soybean were less likely sensitized to other legumes (36.7%–76.7%).

**Table 1:** The frequency of sensitization between 10 legumes in six different legume-allergic patient groups.

When allergic for		% sensitized									
	*n*	Peanut	Soybean	Green pea	Chickpea	Blue lupine	White lupine	Black lentil	Green lentil	Faba bean	White bean
**Peanut**	**30**	90%	76.7%	46.7%	53.3%	66.7%	56.7%	70%	63.3%	40%	60%
**Soybean**	**30**	63.3%	60%	53.3%	43.3%	46.7%	46.7%	50%	43.3%	36.7%	40%
**Green pea**	**30**	76.7%	83.3%	90%	86.7%	83.3%	80%	93.3%	90%	80%	66.7%
**Lupine**	**30**	83.3%	80%	70%	80%	93.3%	83.3%	93.3%	83.3%	70%	63.3%
**Lentil**	**17**	70.6%	82.4%	94.1%	82.4%	94.1%	82.4%	94.1%	88.2%	76.5%	58.8%
**Bean**	**9**	88.9%	100%	100%	100%	100%	88.9%	100%	100%	100%	77.8%

### Allergic patients are always sensitized to other legumes, while peanut, soybean and green pea-allergic patients are not

**Figure 1:**
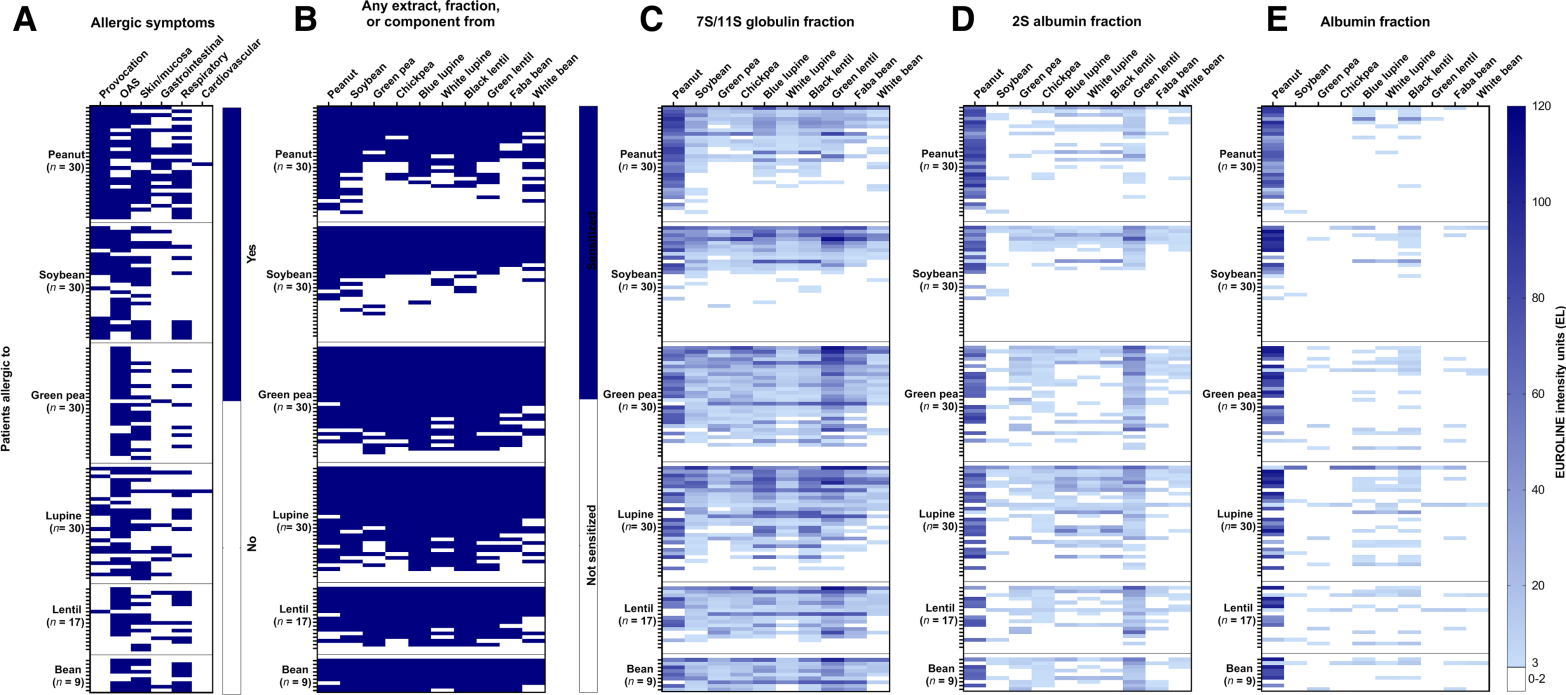
Heat maps showing the clinical details and frequency of co-sensitization for ten legumes in peanut, soybean, green pea, lupine, lentil, bean-allergic patient groups. Severity of the reaction and if diagnosis was based on food challenge (provocation) are shown in (**A**). Patients were ranked positive (blue) when IgE binding was found for either the non-processed or processed extract, the albumin, 2S albumin, or 7S/11S globulin fraction, or an individual protein (**B**). The EUROLINE intensity units were displayed for the 7S/11S globulin fractions (**C**), 2S albumin fractions (**D**), and albumin fractions (**E**), with darker shades of blue indicating a higher intensity of IgE binding. Each row in the different heat maps represent the same patient.

Individual sensitization profiles of the six legume-allergic patient groups to various legume protein extracts are provided in Figure 1. The allergic symptoms for each individual are displayed in Figure 1A. A limited degree of mono-sensitization was found in the soybean (16.7%), peanut (10%), and green pea-allergic (3.3%) patient groups. Co-sensitization for all 10 legumes was frequently seen in all legume-allergic patient groups (peanut (23.3%), soybean (33.3%), green pea (50%), lupine (43.3%), lentil (41.2%), and bean (66.7%) **(**Supplementary Table S3 and Figure 1B)). Remarkably, co-sensitization for less than six legumes was not seen for lupine, lentil, and bean-allergic patients as opposed to peanut, soybean and green pea-allergic patient groups. This indicates that lupine, lentil, and bean-allergic patients were always sensitized to other legumes as well, while peanut and soybean-allergic patients were not. A substantial percentage (20%) of soybean-allergic patients showed no IgE binding to one of the tested soybean extracts. This is most likely caused by the absence of the PR10-protein Gly m 4, an important allergen in soymilk, in the extracts (Supplementary Table S3).

### Co-sensitization is mainly attributable to 7S/11S globulins, and not to (2S) albumins

IgE binding for different protein fractions was further evaluated by heat maps in Figure 1C (7S/11S globulins), Figure 1D (2S albumins), and Figure 1E (albumins). A high frequency of co-sensitization between the 7S/11S globulin fractions of the 10 legumes was observed**,** with IgE binding intensity profiles that resemble the sensitization profile as shown in Figure 1B (any extract, fraction or component). The mean intensity of IgE binding for each of the tested legumes was consistently higher for the 7S/11S globulin fraction compared to the 2S albumin and albumin fraction, except for the peanut 2S albumin fraction. Co-sensitization between 2S albumin fractions was more frequently seen compared to the albumin fractions. The most notable co-sensitization was seen for 2S albumin fraction of peanut and green lentil. 66.7% of the peanut-allergic patients, sensitized to the peanut 2S albumin fraction, were also sensitized to 2S albumin fraction of green lentil. Vice versa, 41.2% of the lentil-allergic patients were also sensitized to the 2S albumin fraction from peanut. The frequency of sensitization for the albumin fraction was in general low, except for peanut.

### High frequency of co-sensitization between individual 7S and 11S globulins of different legumes

**Figure 2:**
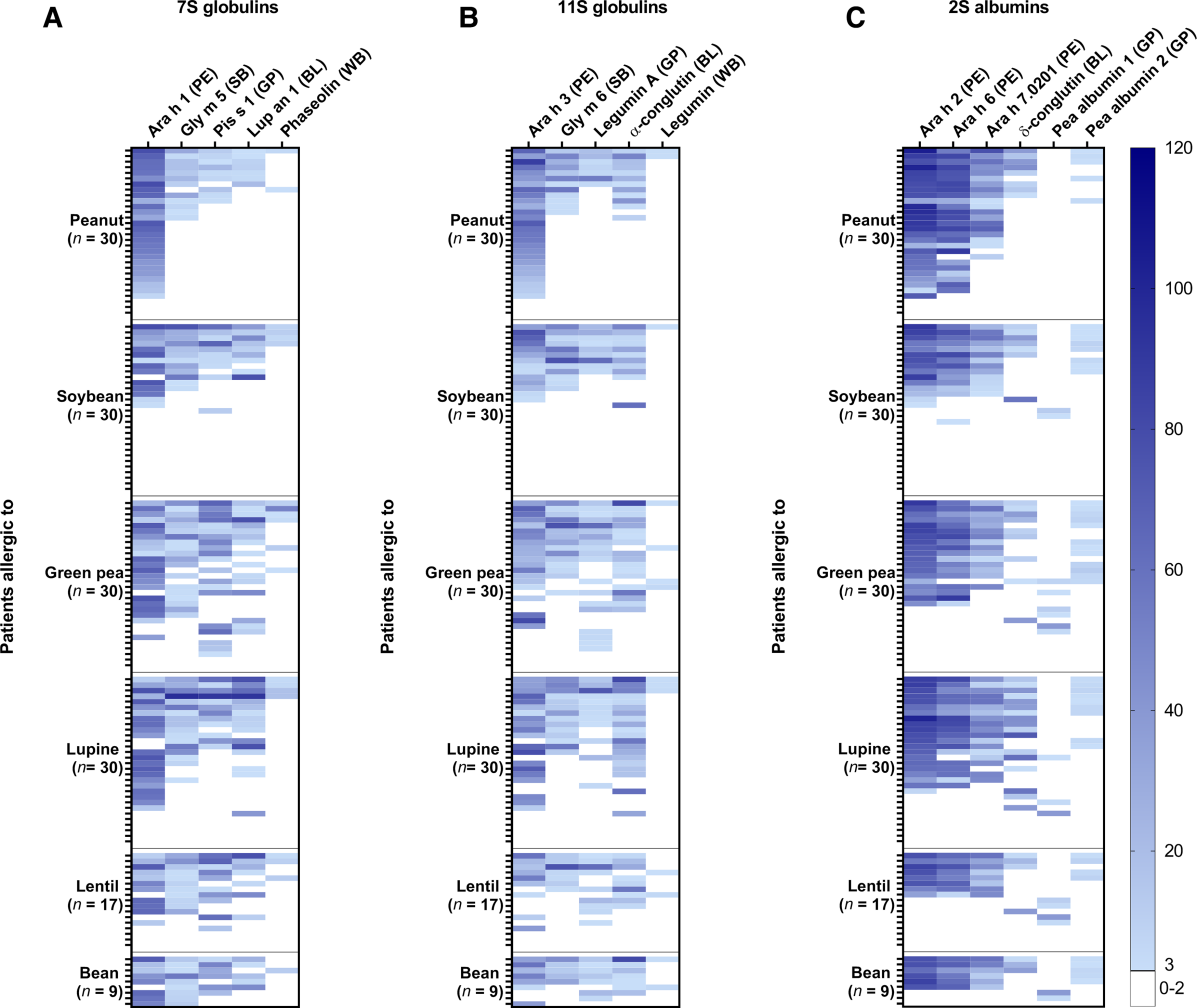
The frequency of sensitization for individual proteins from peanut (PE), soybean (SB), green pea (GP), blue lupine (BL), and white bean (WB) in peanut, soy, green pea, lupine, lentil, and bean-allergic patient groups. (**A**) The intensity of IgE binding was shown for individual 7S globulins, (**B**) 11S globulins, (**C**) and 2S albumins. Darker shades of blue indicated a higher intensity of IgE binding and each row represented the same patient.

The frequency of co-sensitization between individual 7S globulins (Figure 2A), 11S globulins (Figure 2B), and 2S albumins (Figure 2C) was further explored for individual legume proteins. Co-sensitization for individual 7S and 11S globulins was highly prevalent in the soybean, green pea, lupine, lentil, and bean-allergic patient groups, which is consistent with the high co-sensitization between the 7S/11S globulin fractions of the various legumes. Interestingly, co-sensitization for the 7S and 11S globulins was in general frequently seen, with exception to phaseolin and legumin from white bean. The frequency of sensitization for these proteins was low, even in the bean-allergic patient group. Co-sensitization between the peanut 2S albumins and *δ*-conglutin from blue lupine was more frequently seen than between peanut and pea albumin 1, especially in the lupine-allergic patient group. Co-sensitization between the pea albumin 2, the peanut allergens, and blue lupin *δ*-conglutin was observed. 5 out of 7 green pea-allergic patients who were sensitized to pea albumin 1 were mono-sensitized to this 2S albumin. Together, the data indicate that co-sensitization is mainly seen between 7S and 11S globulins and to a lesser extent between 2S albumins.

### Co-sensitizations are often not clinical relevant

**Figure 3:**
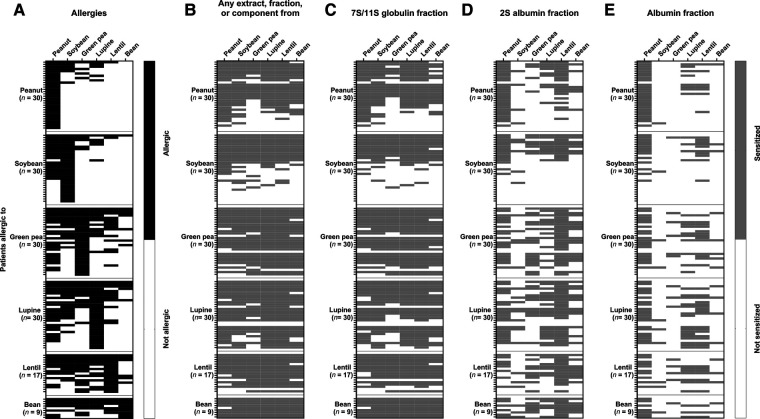
Heat maps showing the frequency of legume allergy and the frequency of sensitization. (**A**) A legume allergy (black cells) was established by either a positive food challenge or a convincing history of legume-allergic reactions combined with a positive IgE test in blood. (**B**) The frequency of sensitization (grey) for any extract, protein fraction, or protein, (**C**) the 7S/11S globulin fraction, (**D**) the 2S albumin fraction, (**E**) and the albumin fraction are also given.

In peanut and soybean-allergic patients, co-allergies for green pea, lupine, lentil and bean were uncommon (≤16,7%), while in green pea, lupine, lentil, and bean-allergic patients a peanut (64.7%–77.8%) or soybean (50%–64.7%) co-allergy was frequently seen (Figure 3, Table 2). A co-allergy for bean (0%–35.3%) was the least common co-allergy in all groups. Conversely, co-allergies to most of the other legumes were frequently (55.6%–77.8%) seen in the bean-allergic group. Furthermore, it is interesting to note that in soybean-allergic patients the frequency of a peanut (63.3%) co-allergy was much higher compared to co-allergy to other legumes (3.3%–16.7%). In all legume-allergic groups, the frequency of co-allergy was less prevalent compared to the frequency of co-sensitization. This indicates that a considerable part of the observed co-sensitizations may be not clinically relevant. The clinical relevance of the sensitization for different protein fractions (Figures 3C–E) is complex to interpret. We therefore calculated the OR for clinical relevant food allergy when sensitized to 7S/11S globulin or 2S albumin fractions. (Table 2). Sensitization for the 7S/11S globulin or the 2S albumin fraction from peanut significantly increased the risk for a peanut co-allergy in soybean, green pea, lupine, and lentil-allergic patients compared to patients that were not sensitized to the 7S/11S globulin or 2S albumin fraction from peanut (OR ranging from 12.89 [95% CI 1.97–84.12] to 94.71 [95% CI 4.39–2041.8]). Peanut and soybean-allergic patients that were sensitized to the 2S albumin fraction from lupine, had a significantly higher chance to have a lupine co-allergy compared to patients that were not sensitized to 2S albumin fraction from lupine (OR 11.18, 95% CI 1.41–88.95 for peanut as well as soybean). However, most of the OR were not statistically significant, indicating that co-sensitization often is not associated with a clinically relevant co-allergy. In conclusion, co-sensitization for 7S/11S globulin or 2S albumin fractions from peanut is associated with clinically relevant co-allergy for peanut in almost all legume-allergic patient groups, whereas co-sensitization for 7S/11S or 2S albumin fractions from other legumes is clinically less relevant.

**Table 2:** Odd ratios (OR) for allergy when sensitized for the 7S/11S globulin and 2S albumin fraction of the respective food.

			OR	
Allergic group	Co-allergy	Prevalence of co-allergy	7S/11S globulin	2S albumin
**Peanut**	Soybean	20% (*n *= 6)	6.7 (0.34–133.59)	1.68 (0.2–14.09)
***n *= 30**	Green pea	13.3% (*n *= 4)	19.59 (0.94–406.44)	2.88 (0.31–26.44)
	Lupine	16.7% (*n *= 5)	6.33 (0.31–127.6)	**11.18** (**1.41**–**88.95)**
	Lentil	3.3% (*n *= 1)	1.62 (0.06–43.25)	1.86 (0.07–49.77)
	Bean	0% (*n *= 0)	–	–
**Soybean**	Peanut	63.3% (*n *= 19)	**18.45** (**2.57**–**132.64)**	**60.64** (**3.03**–**1213.93)**
***n *= 30**	Green pea	16.7% (*n *= 5)	**27.13** (**1.33**–**554.23)**	6.69 (0.99–45.39)
	Lupine	16.7% (*n *= 5)	16.24 (0.81–325.88)	**11.18** (**1.41**–**88.95)**
	Lentil	6.7% (*n *= 2)	5.74 (0.25–130.37)	8.81 (0.39–201.38)
	Bean	3.3% (*n *= 1)	4.83 (0.18–128.79)	10.85 (0.39–298.92)
**Green pea**	Peanut	73.3% (*n *= 22)	**12.89** (**1.97**–**84.12)**	**94.71** (**4.39**–**2041.83)**
***n *= 30**	Soybean	50% (*n *= 15)	3.78 (0.51–28.05)	1.51 (0.25–9.11)
	Lupine	40% (*n *= 12)	0.36 (0.04–3.13)	2.62 (0.61–11.2)
	Lentil	46.7% (*n *= 14)	0.48 (0.06–4.18)	1.55 (0.18–13.4)
	Bean	23.3% (*n *= 7)	5.57 (0.28–112.01)	2.83 (0.52–15.46)
**Lupine**	Peanut	73.3% (*n *= 22)	**21.32** (**3.00**–**151.74)**	**14.49** (**2.28**–**92.12)**
***n *= 30**	Soybean	60% (*n *= 18)	2.49 (0.61–20.18)	0.77 (0.17–3.49)
	Green pea	40% (*n *= 12)	3.35 (0.73–15.38)	1.41 (0.33–6.03)
	Lentil	36.7% (*n *= 11)	0.31 (0.04–2.70)	0.54 (0.08–3.71)
	Bean	16.7% (*n *= 5)	5.34 (0.26–108.26)	6.18 (0.82–46.63)
**Lentil**	Peanut	64.7% (*n *= 11)	6.84 (0.85–54.81)	**31.57** (**1.37**–**725.23)**
***n *= 17**	Soybean	64.7% (*n *= 11)	3.89 (0.39–39.02)	0.26 (0.03–2.58)
	Green pea	82.4% (*n *= 14)	3.00 (0.26–34.33)	0.6 (0.06–5.77)
	Lupine	64.7% (*n *= 11)	0.19 (0.01–4.29)	4.33 (0.51–36.57)
	Bean	35.3% (*n *= 6)	7.8 (0.35–173.98)	6.11 (0.71–52.5)
**Bean**	Peanut	77.8% (*n *= 7)	15.00 (0.39–576.69)	21.67 (0.64–730.03)
***n *= 9**	Soybean	55.6% (*n *= 5)	1.22 (0.02–74.72)	0.33 (0.03–4.26)
	Green pea	77.8% (*n *= 7)	3.00 (0.05–194.75)	2.2 (0.15–33.14)
	Lupine	55.6% (*n *= 5)	1.22 (0.02–74.72)	7.00 (0.49–100.03)
	Lentil	66.7% (*n *= 6)	1.86 (0.03–115.44)	1.86 (0.03–115.44)

Odds ratios with 95% confidence intervals are given for allergy when sensitized for the 7S/11S globulin and 2S albumin fraction of the respective food. Results are shown for each legume-allergic group and for each legume co-allergy. Significant ORs are shown in bold.

### Cross-reactivity occurs between lentil and green pea but not between lentil and peanut

**Figure 4:**
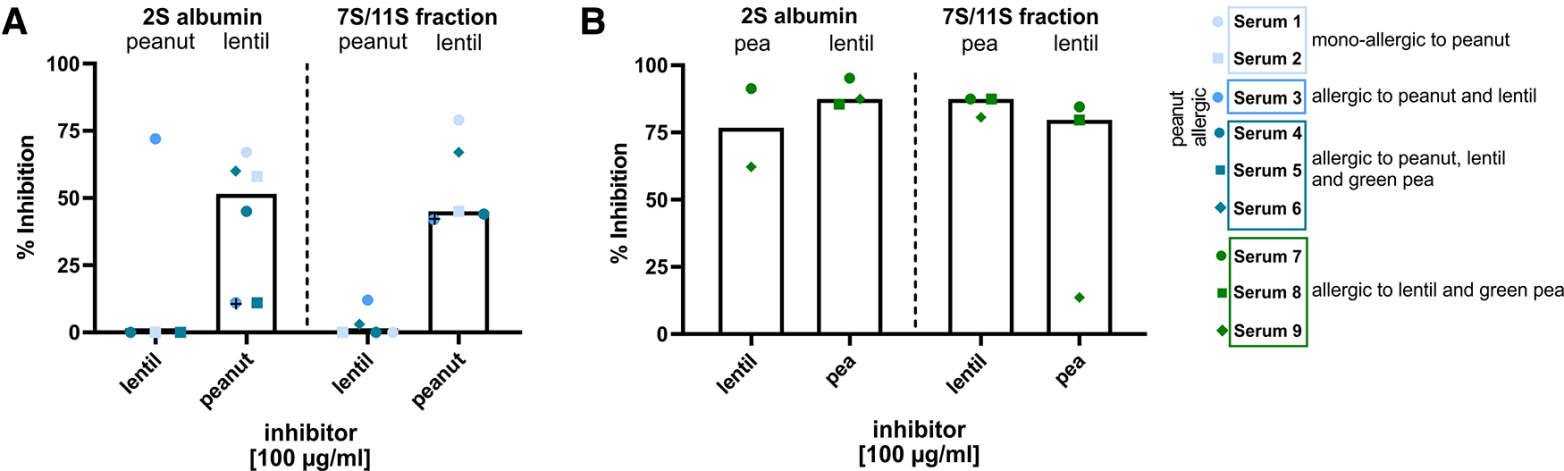
Inhibition of IgE binding to peanut, green pea and lentil 2S albumin and 7S/11S fractions at an inhibitor concentration of 100 µg/ml. (**A**) Cross-inhibition of IgE binding to the 2S albumin and 7S/11S fractions from peanut and lentil in peanut-allergic subjects. (**B**) Cross-inhibition of IgE binding to the 2S albumin and 7S/11S fractions from green pea and lentil in green pea and lentil-allergic subjects.

Inhibition assays were performed as a case study to investigate whether (and to which extent) clinically relevant and irrelevant co-sensitization was caused by cross-reactivity in subpopulation of peanut, green pea and lentil-allergic subjects (Supplementary Table S2, Supplementary Figure S1). Potential cross-reactivity between peanut and lentil fractions (2S albumin and 7S/11S) was examined by cross-inhibition of IgE binding using sera from peanut-allergic and lentil-allergic subjects (Figure 4A, Supplementary Figure S2). An inhibitor concentration of 10 µg/ml was used as a negative control as 100 µg/ml showed inhibition for serum 3 (indicated with a black cross). Pre-incubation with peanut 2S or 7S/11S fractions resulted in some inhibition of IgE binding to the respective lentil fractions (median inhibition 2S albumin: 11%, median inhibition 7S/11S globulin: 67%). Vice versa, no inhibition or only limited inhibition of IgE binding to the 2S albumin and 7S/11S peanut fractions was observed upon pre-incubation with the respective lentil fractions. These observations indicate that sensitization for peanut fractions is, to a limited extent, responsible for co-sensitization for lentil fractions. However, co-sensitization between lentil and peanut is not caused by primary sensitization for lentil fractions. In contrast to peanut-allergic subjects, pre-incubation with lentil and green pea 2S albumin or 7S/11S fractions resulted in an inhibition (79% to 90%) of IgE binding in lentil and pea-allergic subjects (Figure 4B). Thus, clinically relevant co-sensitization between green pea and lentil in subjects co-allergic to green pea and lentil can to a large extent be explained by cross-reactivity.

## Discussion

This study showed that co-sensitization between legumes in different legume-allergic patient groups was highly prevalent. All lupine, lentil, green pea and bean-allergic patients were sensitized to multiple other legumes, in contrast to peanut and soybean-allergic patients that were generally sensitized to a low(er) number of other legumes. The 7S globulin and 11S globulin seed storage proteins were likely major contributors to the observed co-sensitization. The high co-sensitization rate was associated with clinical symptoms in only a relatively small number of patients (e.g., 16.7% of peanut-allergic patients were co-allergic to lupine although 70% of peanut-allergic patients were sensitized to lupine). Remarkably, co-allergy in peanut and soybean-allergic patients was rare, whereas co-allergy in green pea, lupine, lentil, and bean-allergic patients occurred frequently. It should however be noted that blinded oral food challenges towards all studied allergenic sources should be performed to confirm or to exclude the presence of an (co-)allergic reaction. These data underline that knowledge on cross-reactivity and clinical relevance is important to assess the safety for food allergic patients when introducing (novel) foods.

A considerable proportion (>23.3%) of legume-allergic patients were sensitized to all 10 tested legumes and mono-sensitization was only seen in the peanut, soybean and green pea-allergic group to a limited extent (3.3%–16.7%). Interestingly, co-sensitization for a large number (≥6) of legumes occurred less frequently in peanut and soybean-allergic patient groups in contrast to green pea, lupine, lentil, and bean-allergic patient groups. Previous studies also observed that sensitization for multiple legumes was frequent, but most of these studies investigated solely co-sensitization in peanut-allergic patients (6–9, 23). Barnett et al. reported that 38% of peanut-allergic patients had IgE against all tested legumes in their study (peanut, garden pea, soybean, haricot bean, and brown lentil extract) (9). In comparison to the study of Barnett and colleagues, we found that 66.7% of peanut-allergic patients were sensitized to 5 or more legumes. However, comparison of the studies is difficult as the same legumes were not evaluated.

The 7S/11S globulin fraction was the major contributor to the observed co-sensitization between different legumes. This is strengthened by the fact that the IgE binding intensities were generally higher for the 7S/11S fraction compared to the 2S albumin and albumin fraction. It must be noted that residues of proteins may remain in fractions unintentionally, as confirmed by LC-MS and SDS-PAGE analysis (details of the methods are available in the Online Repository and the results can be found in Supplementary Table S4, Supplementary Figure S3, and Supplementary Figure S4). These residues may have influenced IgE binding to the different fractions. However, most proteins were dominant in the expected fractions (e.g., Ara h 2 in 2S albumin fraction). Moreover, IgE-binding results from the individual 2S, 7S, and 11S proteins were comparable to that of the protein fractions which strengthens our results.

Co-sensitization between individual 7S globulins and 11S globulins from different legumes was frequently seen, which explains the substantial role of 7S globulins and 11S globulins in co-sensitization between legumes. The high co-sensitization rates are most likely attributed to the high percentage of amino acid sequence identity (34.2–62.6%) (Supplementary Table S5). A higher percentage of identity (>50%) was reported to be indicative of potential cross-reactivity (24). Interestingly, co-sensitization of the 7S/11S globulin fraction from white bean with other legumes was seen, though co-sensitization was low for the individual 7S and 11S globulin proteins from white bean. The reason for this discrepancy is currently unclear but it could be possible that another (unidentified) 7S/11S globulin, caused the co-sensitization of the white bean 7S/11S globulin fraction and other 7S/11S globulin fractions.

Co-sensitization between 2S albumins was less frequent compared to 7S/11S globulin, though marked co-sensitization was seen between the 2S albumin fraction from peanut and lentil. Unfortunately, we were not able to confirm this using individual proteins, because the 2S albumin(s) from lentil have not yet been identified. Co-sensitization between pea albumin 1 from green pea, a 2S albumin, with other 2S albumins (Ara h 2, 6, 7.0201, *δ*-conglutin) was rarely seen, which could be explained by the low percentage of identity between pea albumin 1 and other 2S albumins (Supplementary Table S5). A low percentage of identity was also found for pea albumin 2, although co-sensitization for this protein and other 2S albumins was frequently seen. This indicates that sequence identity is only partially responsible for the observed co-sensitization. In our opinion pea albumin 1 and 2 are currently incorrectly labelled as 2S albumins as the percentage of identity with other 2S albumins is low and there are large structural differences with other 2S albumins. Remarkably, mono-sensitization was found for pea albumin 1 in 20% of the 30 green pea-allergic patients, indicating the potential value of this protein in diagnosing pea allergy. Our cross-inhibition experiments showed that co-sensitization between green pea and lentil in patients co-allergic to green pea and lentil can to a large extent be explained by cross-reactivity. However, cross-reactivity was relevant to a limited extent for co-sensitization between peanut and lentil fractions. Further inhibition studies are needed to elucidate whether the high rate of co-sensitizations found in this study between the different legumes are caused by cross-reactivity.

This study showed that a large proportion of the co-sensitizations were not clinically relevant. Especially in the peanut and soybean-allergic patient groups, we noticed that co-allergy with other legumes was rarely seen. The frequencies of co-allergy might be slightly underestimated because the data in our study was not collected systemically with a standardized questionnaire, but using the available data from the electronic patient file from routine care. However, we think that collecting the data in this manner is not a likely explanation for the clinically irrelevant sensitizations. In the green pea, lupine, lentil and bean-allergic patient group, co-allergy with peanut was almost inevitable (74.7%–77.8%) and co-allergy with other legumes appeared frequently (up to 82.4%). The low frequency of clinical allergy in patients sensitized to legumes was previously reported by others (6, 7, 23). For example, Bock et al. showed that of 32 peanut-allergic patients 17 were sensitized to soybean and 15 to pea, but a clinically relevant reaction was seen for both foods in only one patient (23). Our study underlines that IgE binding only leads to clinical symptoms in a minority of legume-allergic patients, which is most prominently seen in peanut-allergic patients. It seems that sensitization (and allergy) caused by peanut, and to a lesser extent by soybean, follows a distinctly different pattern in regard of co-sensitization and co-allergy compared to the other evaluated legumes.

In conclusion, we showed that co-sensitization between legumes in different legume-allergic patient groups is frequently seen, but that large proportions of these co-sensitizations were not clinically relevant. The observed co-sensitization could be mainly attributed to the 7S and 11S globulins, although 2S albumins could also be partly responsible. Legumes are an attractive sustainable protein source, but cross-reactive allergic reactions in the already legume-allergic population cannot be excluded as co-sensitization and, to a lesser extent, co-allergy was observed in multiple legume-allergic patient groups. Future studies, including larger patient groups are needed to confirm these findings.

## Data Availability

The raw data supporting the conclusions of this article will be made available by the authors, without undue reservation.
