# Supplementary material for: Co-sensitization between legumes is frequently seen, but variable and not always clinically relevant
Source: Front Allergy. 2023 Mar 16;4:1115022. doi: 10.3389/falgy.2023.1115022 (PMC10060518; doi:10.3389/falgy.2023.1115022)
Supplement: Supplementary file 1 [file Datasheet1.docx]

Supplementary Material

**Table S1.** Patient details.

|  | | **Peanut** | **Soybean** | **Green pea** | **Lupine** | **Lentil** | **Bean** |
| --- | --- | --- | --- | --- | --- | --- | --- |
| **Mean age (±SD)** | | 27.8 (7.9) | 34.1 (14.9) | 31.7 (11.0) | 33.6 (12.5) | 36.5 (10.4) | 31,9 (11.6) |
| **Sex (% male)** | | 30.0% | 23.3% | 43.3% | 36.7% | 47.1% | 44.4% |
| **Inclusion based on** | |  |  |  |  |  |  |
| Positive provocation (%) | 100% | 63.3% | 0% | 40.0% | 5.9% | 0% |  |
| Convincing history and positive IgE test (%) | 0% | 36.7% | 100% | 60.0% | 94.1% | 100% |  |
| **Symptoms** | |  |  |  |  |  |  |
| OAS (%) | 73.3% | 73.3% | 86.7% | 76.7% | 70.6% | 77.7% |  |
| Skin/mucosa (%) | 76.7% | 60.0% | 46.7% | 50% | 47,10% | 77.7% |  |
| Gastrointestinal (%) | 43.3% | 10.0% | 3.3% | 23.3% | 17.6% | 11.1% |  |
| Respiratory (%) | 66.7% | 26.7% | 20% | 26.7% | 47,10% | 77.7% |  |
| Cardiovascular (%) | 3.3% | 0% | 0% | 3.3% | 0% | 0% |  |

**Table S2.** Subpopulation of legume-allergic patients for the inhibition assay.

|  | |  | **Allergy** |  |
| --- | --- | --- | --- | --- |
| **Serum** | | **Peanut** | **Green Pea** | **Lentil** |
| S1 |  | X |  |  |
| S2 |  | X |  |  |
| S3 |  | X |  | X |
| S4 |  | X | X | X |
| S5 |  | X | X | X |
| S6 |  | X | X | X |
| S7 |  |  | X | X |
| S8 |  |  | X | X |
| S9 |  |  | X | X |

**

**Figure S1.** Sensitization patterns of peanut and green pea-allergic patients used for inhibition assays.

**Table S3**. Percentage co-sensitization in peanut, soybean, green pea, lupine, lentil, and bean-allergic patients for any extract, protein fraction or individual component.

| **Legume allergy** | **Sensitized to number of legumes** | | | | |
| --- | --- | --- | --- | --- | --- |
|  | **0** | **1** | **2-5** | **6-9** | **10** |
| **Peanut** | 3.3% | 10% | 33.3% | 30% | 23.3% |
| **Soybean** | 20% | 16.7% | 20% | 10% | 33.3% |
| **Green pea** | 3.3% | 3.3% | 3.3% | 40% | 50% |
| **Lupine** | 6.7% | 0% | 6.7% | 43.3% | 43.3% |
| **Lentil** | 5.9% | 0% | 5.9% | 47.1% | 41.2% |
| **Bean** | 0% | 0% | 0% | 33.3% | 66.7% |

**Table S3**. Presence of 2S albumins, and 7S and 11S globulins in the protein fractions.

| **Peanut** | **Allergen name** | **Accession number** | **2S/7S/11S** | **Albumin** | **2S albumin** | **7S/11S globulin** |  |
| --- | --- | --- | --- | --- | --- | --- | --- |
|  | Ara h 1 | P43238, E5G076 | 7S | ++ | ++ | +++ |  |
|  | Ara h 2 | Q6PSU2 | 2S | - | +++ | ++ |  |
|  | Ara h 3 | O82580 | 11S | - | - | ++ |  |
|  | Ara h 6 | Q647G9, A5Z1Q5 | 2S | + | ++ | ++ |  |
|  | Ara h 7.0201 | B4XID4 | 2S | - | + | + |  |
| **Soybean** | Gly m 4 | P26987 | - | - | - | - |  |
|  | Gly m 5 | O22120 | 7S | - | ++ | +++ |  |
|  | Gly m 6 | P04776 | 11S | - | ++ | +++ |  |
|  |  | P04405 |  |  |  |  |  |
|  | Gly m 8 | P19594 | 2S | + | +++ | +++ |  |
|  | Basic 7S globulin 1 | P13917 | 7S | - | + | ++ |  |
|  | Basic 7S globulin 2 | Q8RVH5 | 7S | - | + | ++ |  |
| **Green pea** | Pea albumin 1 | P62926, P62928, | 2S | + | + | + |  |
|  |  | P62930 |  |  |  |  |  |
|  | Pea albumin 2 | P08688 | 2S | + | ++ | - |  |
|  | Pis s 1 | Q702P1 | 7S | - | - | ++ |  |
|  | Pis s 2 | P13918,  Q9M3X6 | 7S | + | ++ | ++ |  |
|  | Legumin A | P02857 | 11S | + | - | +++ |  |
| **Chickpea** | Vicilin-like | A0A1S2Y087 | 7S | + | ++ | ++ |  |
|  | Legumin-like | A0A1S2XTK6 | 11S | + | +++ | +++ |  |
|  | 2S albumin-like | A0A1S2XDF0 | 2S | + | ++ | + |  |
| **Blue lupine** | Lup an 1 | B8Q5G0 | 7S | + | ++ | +++ |  |
|  |  | B0YJF8 |  |  |  |  |  |
|  |  | F5B8W3 |  |  |  |  |  |
|  | Conglutin α | F5B8V6 | 11S | + | ++ | ++ |  |
|  |  | F5B8V7 |  |  |  |  |  |
|  | Conglutin δ | F5B8W8 | 2S | + | +++ | ++ |  |
|  |  | F5B8X0 |  |  |  |  |  |
|  | Conglutin γ | Q42369 | 7S | - | - | - |  |
| **White lupine** | Conglutin β | Q53HY0 | 7S | + | +++ | +++ |  |
|  | Conglutin δ | Q333K7 | 2S | + | +++ | + |  |
|  | Conglutin γ | Q9FSH9 | 7S | + | + | + |  |
|  | Legumin-like protein | Q53I54 | 11S | - | + | + |  |
| **Black lentil** | Len c 1 | Q84UI1 | 7S | + | ++ | ++ |  |
|  | Convicilin | Q9M3X8 | 7S | + | ++ | ++ |  |
| **Green lentil** | Len c 1 | Q84UI1 | 7S | - | + | + |  |
|  | Convicilin | Q9M3X8 | 7S | - | + | + |  |
| **Faba bean** | Legumin | Q43673 | 11S | ++ | ++ | ++ |  |
|  | Vicilin | P08438 | 7S | + | ++ | ++ |  |
|  | Convicilin | B0BCL8 | 7S | + | ++ | +++ |  |
|  | Legumin type B | P05190 | 11S | - | + | ++ |  |
| **White bean** | Phaseolin, alpha-type | X5CHV9 | 7S | - | ++ | + |  |
|  | Phaseolin, beta-type | P02853 | 7S | - | ++ | - |  |
|  | Legumin | F8QXP7 | 11S | - | - | +++ |  |

2S albumins, and 7S and 11S globulins were identified in the protein fractions. The presence of these proteins was investigated using LC-MS and the PSM was used as a measure of protein abundance of a specific fraction. For simplicity, we only included the accession number of the identified (intact) proteins with the highest PSM value. The PSM of the proteins in the different fractions were compared for each legume. The difference between +++ and ++ is a 3-fold change in PSM value, the difference between ++ and + is again a 3-fold change in PSM value, and a – indicates that the protein was not identified in a specific fraction.

**
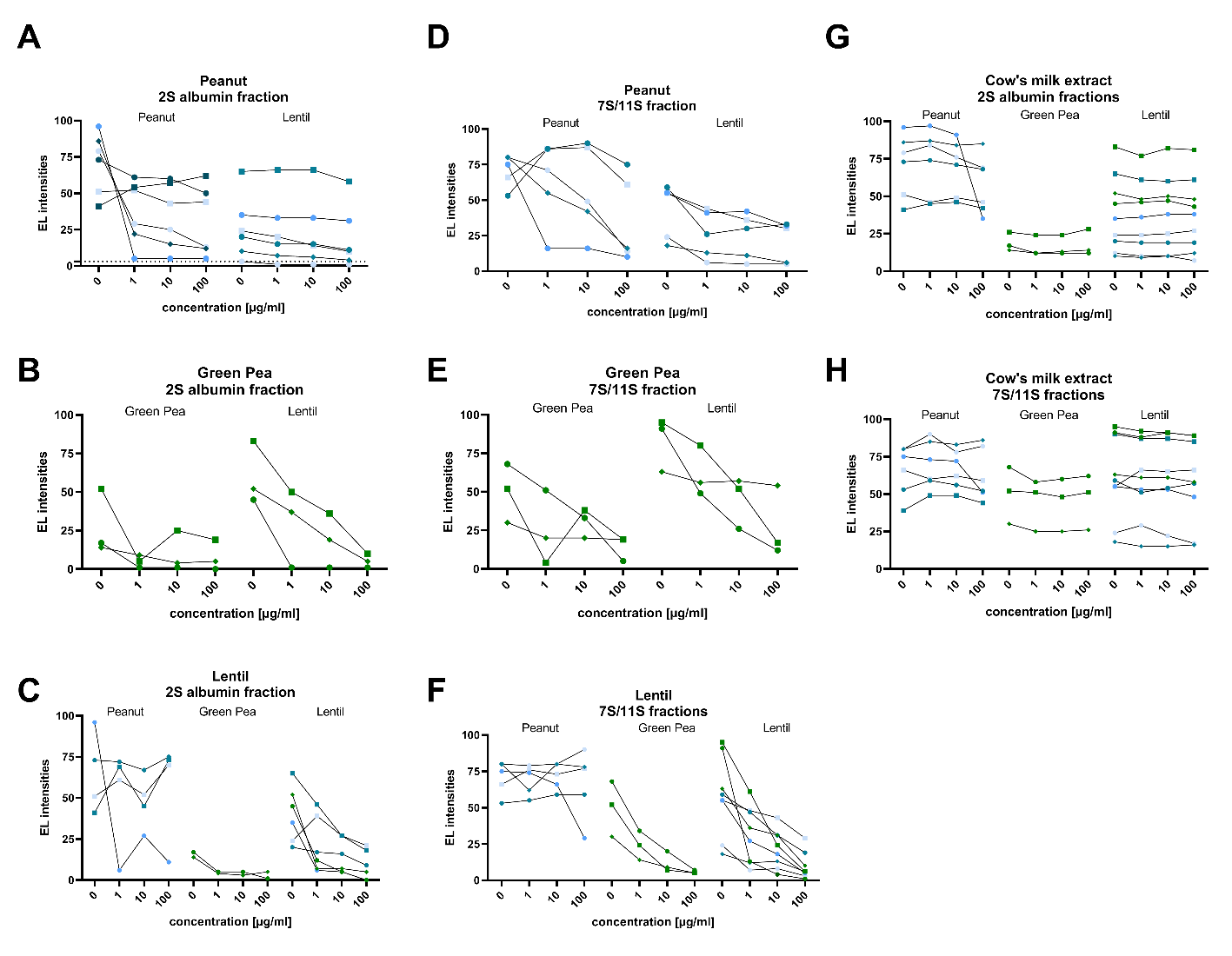
**

**Figure S2**. Inhibition of IgE binding to the 2S albumin and 7S/11S fractions of peanut, green pea and lentil in a concentration dependent manner expressed as decrease in EL-intensities. (**A-C**) Inhibition of sgE binding to 2S albumin fractions by pre-incubation with 2S albumin fractions; (**D-F**) Inhibition of IgE binding to 7S/11S fractions by pre-incubation with 7S/11S fractions; (**G-H**) Inhibition of IgE binding to 2S albumin and 7S/11S fractions by pre-incubation with CME (negative control). Color coding of legume-allergic patients; light blue: peanut-allergic subjects (dot: serum 1, square: serum 2); blue: peanut and lentil-allergic subjects (dot: serum 3), dark blue: peanut, lentil and green pea-allergic subjects (dot: serum 4, square: serum 5, triangle: serum 6); green: green pea and lentil-allergic subjects (dot: serum 7, square: serum 8, triangle: serum 9).


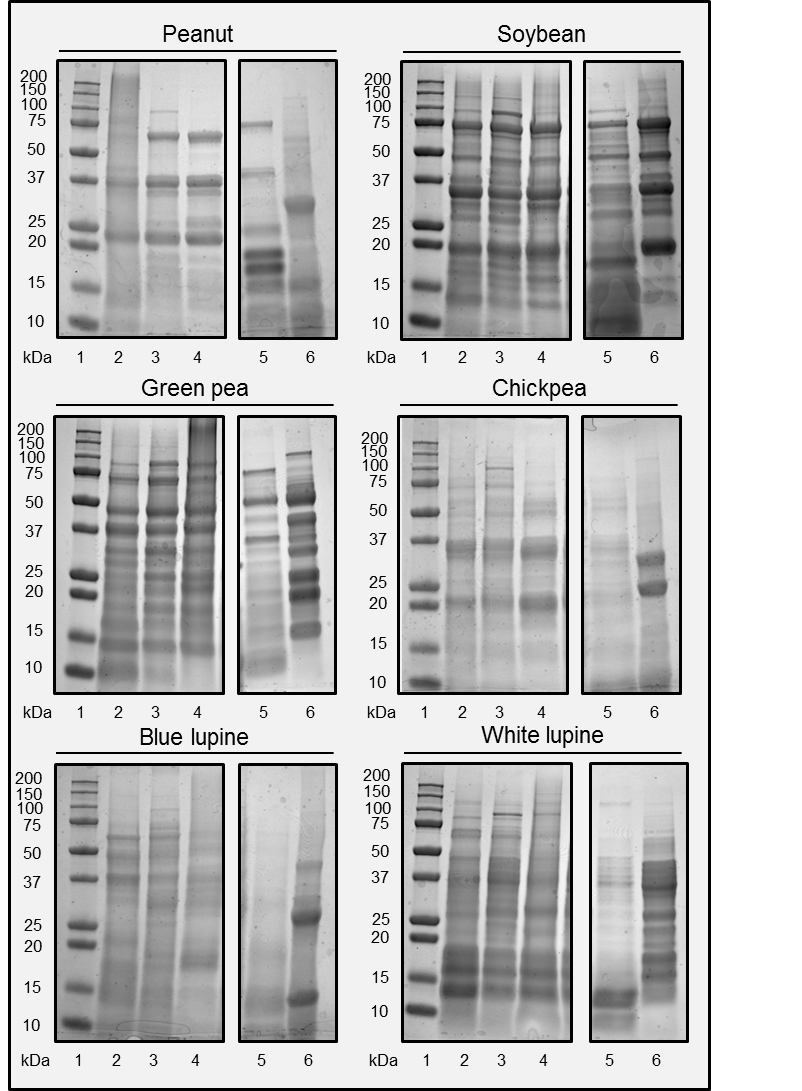


**Figure S3.** SDS-PAGE of total extracts and protein fractions of peanut, soybean, green pea, chickpea, and blue and white lupine. Processed (lane 2) and non-processed (lane 3) total extracts, and 7S/11S globulin (lane 4), 2S albumin (lane 5) and albumin (lane 6) fractions. Lane 1 contained the molecular weight markers. InstantBlue™ (Coomassie) gel staining was used to visualize the protein bands. Information on Molecular Weight of allergens can be found in **Table S4**.

*
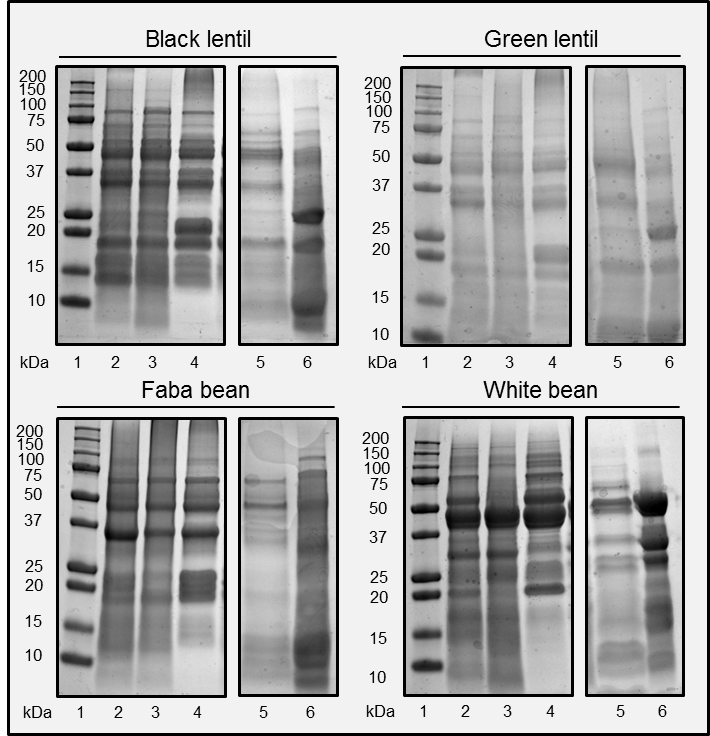
*

**Figure S4**. SDS-PAGE of total extracts and protein fractions of black and green lentil, and faba and white bean. Processed (lane 2) and non-processed (lane 3) total extracts, and 7S/11S globulin (lane 4), 2S albumin (lane 5) and albumin (lane 6) fractions were evaluated. Lane 1 contained the molecular weight markers. InstantBlue™ (Coomassie) gel staining was used to visualize the protein bands. Information on Molecular Weight of allergns can be found in **Table E4**.

**Table S4**. Molecular weight of legume proteins under reducing conditions.

| **Legume** | **Allergen name** | **Accesion number** | **Molecular weight (kDa)** |
| --- | --- | --- | --- |
| Peanut | Ara h 1 | P43238 | 63 |
| Peanut | Ara h 2 | Q6PSU2 | 18, 20 |
| Peanut | Ara h 3 | O82580 | 65, 40, 25 |
| Peanut | Ara h 6 | Q647G9 | 15 |
| Peanut | Ara h 7.0201 | B4XID4 | 15 |
| Soybean | Gly m 5 | O22120,  P11827,  P25974 | 66, 45 |
| Soybean | Gly m 6 | P04776,  P04405,  P11828,  Q9SB11,  Q7GC77 | 40, 20 |
| Green pea | Pis s 1 | Q702P1 | 44 |
| Green pea | Legumin A | P02857 | 22-28, 40 |
| Green pea | Pea albumin 1 | P62926 | 6 |
| Green pea | Pea albumin 2 | P08688 | 28 |
| Blue lupine | Lup an 1 | B8Q5G0 | 55-61 |
| Blue lupine | α-conglutin | F5B8V8 | 65, 50, 40, 20 |
| Blue lupine | δ-conglutin | F5B8W8 | 13, 5 |
| White bean | Phaseolin | Q43632 | 45 |
| White bean | Legumin | F8QXP7 | 75, 50, 40, 25 |

**Table S5.** Percentage identity between the 2S albumins, and 7S and 11S globulins based on BLASTP tool.

| **2S albumins** |  |  |  | |  | |  | |  | |  | | | |  |
| --- | --- | --- | --- | --- | --- | --- | --- | --- | --- | --- | --- | --- | --- | --- | --- |
| Legume | UniProt identifier | Allergen name | **Ara h 2** | **Ara h 6** | | **Ara h 7.0201** | | **δ-conglutin** | | **Pea albumin 1** | | **Pea albumin 2** | |  |  |
| Peanut | Q6PSU2 | **Ara h 2** |  | 55.6% | | 46.8% | | ns | | ns | | ns | |  |  |
| Peanut | Q647G9 | **Ara h 6** | 55.2% |  | | 50.8% | | ns | | ns | | ns | |  |  |
| Peanut | B4XID4 | **Ara h 7.0201** | 49.6% | 51.5% | |  | | 40.3% | | ns | | ns | |  |  |
| Blue lupine | F5B8W8 | **δ-conglutin** | 36.3% | 40.7% | | 44.4% | |  | | ns | | ns | |  |  |
| Pea | P62926 | **Pea albumin 1** | ns | ns | | ns | | ns | |  | | ns | |  |  |
| Pea | P08688 | **Pea albumin 2** | ns | ns | | ns | | ns | | ns | |  | |  |  |
| **7S globulins** |  |  |  |  | |  | |  | |  | | |  |  |  |
| Legume | UniProt identifier | Component name | **Ara h 1** | **Gly m 5** | | **Pis s 1** | | **Lup an 1** | | **Phaseolin** | | |  |  |  |
| Peanut | P43238 | **Ara h 1** |  | 49.0% | | 53.1% | | 53.8% | | ns | | |  |  |  |
| Soybean | O22120 | **Gly m 5** | 45.3% |  | | 54.4% | | 53.1% | | ns | | |  |  |  |
| Pea | Q702P1 | **Pis s 1** | 52.5% | 55.1% | |  | | 59.0% | | ns | | |  |  |  |
| Blue lupine | B8Q5G0 | **Lup an 1** | 47.2% | 46.9% | | 59.1% | |  | | ns | | |  |  |  |
| White bean | Q43632 | **Phaseolin** | 40.4% | 52.9% | | 44.7% | | 42.6% | |  | | |  |  |  |
| **11S globulins** |  |  |  |  | |  | |  | |  | | |  |  |  |
| Legume | UniProt identifier | Component name | **Ara h 3** | **Gly m 6** | | **Legumin A** | | **α-conglutin** | | **Legumin** | | |  |  |  |
| Peanut | O82580 | **Ara h 3** |  | 55.5% | | 50.9% | | 43.3% | | 34.5% | | |  |  |  |
| Soybean | P04776 | **Gly m 6** | 55.9% |  | | 61.3% | | 49.0% | | 43.9% | | |  |  |  |
| Pea | P02857 | **Legumin A** | 60.3% | 60.3% | |  | | 46.5% | | 34.2% | | |  |  |  |
| Blue lupine | F5B8V8 | **α-conglutin** | 42.2% | 47.0% | | 41.2% | |  | | 44.5% | | |  |  |  |
| White bean | F8QXP7 | **Legumin** | 34.9% | 42.6% | | 43.0% | | 62.6% | |  | | |  |  |  |
